# Idiopathic intracranial hypertension and pregnancy: outcomes in previously shunted women

**DOI:** 10.1007/s00701-026-07017-9

**Published:** 2026-08-29

**Authors:** Manolis Polemikos, Hans E. Heissler, Elvis J. Hermann, Dirk Scheinichen, Constantin S. von Kaisenberg, Peter Hillemanns, Joachim K. Krauss

**Affiliations:** 1 Department of Neurosurgery, Hannover Medical School, MHH, Carl-Neuberg-Str. 1, 30625 Hannover, Germany; 2 Department of Gynaecology & Obstetrics, Hannover Medical School, MHH, Hannover, Germany; 3 Department of Anaesthesiology and Intensive Care Medicine, Hannover Medical School, MHH, Hannover, Germany

**Keywords:** Idiopathic intracranial hypertension, Intracranial pressure, Pregnancy, Pseudotumor cerebri syndrome, Ventriculoperitoneal shunt

## Abstract

**Objective:**

Idiopathic intracranial hypertension (IIH) typically affects obese women of reproductive age. Ventriculoperitoneal (VP) shunting is an established surgical treatment for severe or medically refractory cases. There is limited knowledge regarding the management of these patients when they become pregnant. Here, we report our multidisciplinary experience in this subgroup of IIH.

**Methods:**

Our database on IIH was retrospectively screened for women who were diagnosed and treated with a VP shunt from 2005 to 2025. We analyzed onset of IIH in relation to pregnancy, number of pregnancies resulting in live births before and after VP shunting, and the mode of delivery.

**Results:**

Overall 61 women with IIH underwent VP shunting during the study period. Nine women had 12 pregnancies resulting in live births at a median of 40.5 months after VP shunting (range 6–108). Onset of IIH was not related to the occurrence of pregnancies in all but one patient. 9/12 pregnancies resulted in vaginal delivery. Adjustment of the shunt valve was not required in these patients. One patient experienced progressive visual loss refractory to acetazolamide treatment during the first trimester. VP shunting at 11 weeks of gestation facilitated stabilization of her visual symptoms and delivery via elective Caesarean section. There were no peripartum cases of infection or malfunction of the shunt system.

**Conclusions:**

The complexity of IIH during pregnancy requires a multidisciplinary approach in order to achieve an optimal outcome. In our cohort of previously shunted IIH patients with programmable valves and gravitational units, neither the occurrence of a pregnancy nor a vaginal delivery increased the risk for disease recurrence or deterioration. In severe cases, VP shunting remains a treatment option during pregnancy when clinically indicated.

## Introduction

Pseudotumor cerebri syndrome (PTCS) encompasses a spectrum of disorders characterized by elevated intracranial pressure (ICP) with normal cerebrospinal fluid (CSF) composition in the absence of hydrocephalus, and without mass or structural lesions [10, 11]. PTCS includes both primary and secondary forms. While no underlying cause for the elevated ICP can be identified in primary PTCS, secondary PTCS is attributable to an underlying condition [11]. Idiopathic intracranial hypertension (IIH) is the most common primary form of PTCS, which typically affects obese women of childbearing age. The incidence in this subgroup is up to 20 times higher than in the general population [6, 17]. Headache is the most frequent presenting symptom, and papilledema, when present, can result in a decline of vision if left untreated [19].

Given its epidemiology, pregnancy-related considerations are highly relevant in clinical practice. While pregnancy itself does not appear to worsen the long-term prognosis of IIH, physiological changes such as weight gain, increased abdominal pressure, and hormonal changes may exacerbate symptoms [23]. In severe or medically refractory cases, ventriculoperitoneal (VP) shunting is a well-established treatment option [12, 13, 19]. Despite the widespread use of shunts in IIH, data on disease and pregnancy outcomes in women who underwent VP shunting for IIH remain limited. Available data are mostly restricted to isolated case reports or small heterogeneous series lacking systematic evaluation. Concerns in this population include the potential for shunt malfunction during pregnancy, increased intracranial pressure during labor, and optimal delivery mode [7].

Here, we address this gap by evaluating pregnancy and delivery outcomes in a cohort of women with IIH who underwent VP shunting either prior to conception or during pregnancy. We analyze maternal and neonatal outcomes, shunt stability and delivery mode in a multidisciplinary setting.

## Methods

### Study population and data collection

We performed a retrospective observational study based on our prospectively maintained database of women with IIH who underwent VP shunting in our department between January 2005 and May 2025. Women who had at least one pregnancy resulting in live birth after VP shunt implantation or underwent VP shunt implantation during pregnancy were included in the primary outcome analysis. The diagnosis of IIH was established according to the Friedman-Jacobson modification of the Dandy-criteria and their revision since 2013[9, 11]. In addition, in 59 patients the diagnosis was supported by continuous ICP monitoring via an epidural sensor implanted over the frontal area [21, 22]. Monitoring was performed prior to VP shunting and typically lasted 1–2 days, with particular focus on nocturnal recordings.

At our institution, VP shunt implantation is considered in patients with progressive visual deterioration or persistent papilledema despite medical therapy, intolerance to pharmacological treatment, or persistent disabling symptoms in the presence of documented intracranial hypertension.

The surgical procedure of VP shunt placement was performed according to an institutional guideline, involving whole head shaving, a precoronal burr hole and a subxiphoidal midline abdominal incision (mini laparotomy). All patients underwent implantation of a programmable valve and an integrated gravitational unit [24]. Between 2005 and 2015, proGAV valves (Miethke, Potsdam, Germany) were used, whereas proGAV 2.0 valves were implanted thereafter. Insertion of the ventricular catheter was aided by neuronavigation as previously described [13]. Accurate positioning of the VP shunt was confirmed on early postoperative CT scan and in shunt radiograms [8].

Demographic data which were collected included age at surgery and body mass index (BMI) at the time of shunt implantation. Patients were classified as being of reproductive age using the World Health Organization (WHO) definition of 15–49 years [27]. The gynecological history was reviewed to evaluate fertility status. Patients with known fertility-limiting history (e.g., hysterectomy) were classified as non-fertile, even if within the WHO reproductive age range. Additional pregnancy-related variables included timing of IIH onset in relation to pregnancy, number of pregnancies resulting in live births before and after shunt placement, mode of delivery, and peripartum complications. Information regarding initial opening pressure and any valve adjustments was also recorded. Pregnancies before VP shunting were collected to evaluate reproductive history.

According to institutional guidelines, ethical approval and informed consent were not required for this retrospective analysis of anonymized data.

### Statistical analysis

Descriptive statistics, including mean, median, ranges, frequencies and percentages, were used for patient demographics and clinical characteristics.

## Results

### Patient characteristics

A total of 61 women underwent VP shunting for PTCS during the study period, with a median age at surgery of 33 years (range 18–67) and a median BMI of 36.3 kg/m^2^ (range 19.1–52.5). The average interval between diagnosis of IIH and VP shunting was 14 months (range 0–193). The initial setting of the programmable shunt valve was typically 6 cmH₂O, with initial settings ranging from 6 to 16 cmH_2_O in our cohort. The demographic and clinical characteristics are summarized in Table 1.

**Table 1:** Demographic and clinical characteristics of 61 women with IIH

Variable	Value**
Women of reproductive age (15–49 years)* at VP-shunting	49 (80.3%)
Patients aged ≤ 35 years	34 (55.7%)
Median age at VP shunting in years	33 (range: 18–67)
Median BMI at VP shunting in kg/m^2^	36.3 (range: 19–52.5)
Median diagnosis to VP shunting interval in months	15.5 (range 0–193)
Women with live births before VP shunting	28
Live births prior VP shunting	63
Women with live births after VP shunting	9
Live births after VP shunting	12
Median interval VP shunting to delivery in months	40.5 (range: 6–108)
Follow-up duration after VP shunting in months	Median: 46 months; mean: 64.1 months (range: 3–245)

*After excluding patients with a known fertility-limiting history

**Values reported as n unless otherwise indicated

### Pregnancy cohort and delivery outcomes

Among 61 women who underwent VP shunting, 9 women had a total of 12 live births after VP shunt implantation. One of these women underwent VP shunt implantation during pregnancy due to rapid visual deterioration. This case is described in detail below. In 5 of these 9 cases, the post-shunt pregnancy represented the patient’s first pregnancy. The median interval between shunt implantation and delivery was 40.5 months (range 6–108 months).

Pregnancy and delivery outcomes were uneventful in all instances. No patient experienced symptoms suggestive of shunt malfunction or elevated ICP necessitating adjustment of the shunt valve. Nine of 12 post-shunt pregnancies resulted in full-term vaginal deliveries without shunt-related complications. Among the remaining three pregnancies one elective Cesarean section was performed due to persistent visual symptoms and maternal concern about further deterioration. The other two Cesarean deliveries were performed for obstetric indications unrelated to IIH or the VP shunt.

Compared with the overall VP shunt cohort, women who subsequently became pregnant were younger at the time of shunt implantation (median 29 vs 33 years) and had a lower median BMI (29.7 vs 36.3 kg/m^2^).

### Temporal relationship between IIH and pregnancy

The diagnosis of IIH was not temporally related to pregnancy in all but one patient. In this case, the patient developed IIH symptoms during the third trimester, and the diagnosis was confirmed postpartum, leading to VP shunt placement 6 months later.

### Reproductive status

In our cohort 51/61 (84%) of the women met the WHO definition of reproductive age (15–49 years) at the time of shunt implantation. Among these, two women had undergone a hysterectomy before shunting and were excluded from further fertility analysis, leaving 49 potentially fertile patients.

Prior to VP shunting, 28 women had delivered a total of 63 children. Four of these women subsequently had an additional pregnancy after VP shunt implantation. At the time of shunt surgery, 20 of these women were within the reproductive age range.

### Gynecological history

Several women in our cohort had gynecological comorbidities or had undergone interventions potentially affecting fertility. Three patients had undergone hysterectomy during reproductive age prior to VP shunt implantation and were therefore considered infertile; one of them had given birth to two children before the procedure. Other relevant gynecological conditions included polycystic ovary syndrome with ovarian cyst excision (*n* = 4), adnexitis (*n* = 2) and a congenital vaginal septum, which was surgically corrected. Notably, three shunted women experienced a total of four pregnancies following gynecological surgery, indicating that favorable reproductive outcomes in women with IIH are achievable in selected cases after such interventions.

### Representative Case: continuous ICP monitoring and VP shunting during pregnancy for medically refractory IIH

This 27-year-old woman had a suspected diagnosis of IIH in early 2019. At 7 weeks’ gestation (gravida 3, para 1) she was referred for further evaluation due to progressively worsening headaches and visual decline despite treatment with acetazolamide (1500 mg daily) and repeated lumbar punctures (opening pressures between 30–40 cmH₂O). Ophthalmologic examination confirmed papilledema and visual deterioration. An epidural sensor (Neurodur®, Raumedic, Münchberg, Germany) was implanted over the frontal area via a precoronal burr hole under local anesthesia for continuous ICP monitoring [21, 22]. Nocturnal recording during sleep revealed raised ICP (median 22 mmHg). ICP oscillations occurred in the form of B-waves and ramp-like B-waves as well as complex wave forms reaching up to 40 mmHg. Subsequent to ICP monitoring, a VP shunt was implanted using an electromagnetic-guided neuronavigation system (Medtronic, Minneapolis, MN, USA). There was immediate improvement of her headaches and of visual decline after shunting. Later during her pregnancy, symptoms of overdrainage prompted valve adjustment from 6 to 8 cmH₂O. She gave birth to a healthy female infant after an elective Cesarean section.

## Discussion

IIH predominantly affects young women of reproductive age, making pregnancy-related considerations highly relevant in clinical practice. While pregnancy itself does not appear to trigger IIH or worsen long-term visual outcomes [16, 25] recent population-based data indicate that affected individuals may be at increased risk for obstetric complications such as preterm delivery, hypertensive disorders, and postpartum wound complications compared to the general obstetric population [3].

Although the safety of pregnancy in women with VP shunts has been addressed in hydrocephalus cohorts [1, 7], data specifically focusing on women with IIH who became pregnant after shunting or required CSF diversion during pregnancy remain limited. Karimaghaei et al. [15] reported a total of 12 CSF diversion procedures in pregnant IIH patients up to 2018, including 11 lumboperitoneal and one ventriculoperitoneal shunt. A more recent systematic review found that only 3.9% of 178 reported IIH pregnancies involved CSF shunting [20]. Yet no study to date has systematically assessed outcomes in women with IIH following VP shunt placement.

Our study shows that pregnancy and delivery do not adversely affect shunt function or disease stability in women with IIH. VP shunting in our cohort was performed according to a uniform protocol using programmable valves and gravitational units, which allow opening pressure adjustments and prevent overdrainage, particularly relevant during pregnancy [12, 24].

While increased intra-abdominal pressure can be a risk factor for VP-shunt dysfunction [18], our data indicate that the physiological abdominal pressure changes associated with pregnancy do not appear to compromise shunt function in previously shunted women with programmable valves and gravitational units. In our cohort, we observed no evidence of shunt malfunction, no need for valve adjustment, or adverse maternal and neonatal outcomes during pregnancy or delivery. These results align with the broader hydrocephalus literature, showing that while antenatal concerns about shunt malfunction were common, actual mechanical failures were rare, and most pregnancies proceeded without incidents [7].

One patient in our cohort required shunt implantation during the first trimester due to progressive visual deterioration refractory to pharmacological therapy. This case highlights that, although most IIH cases do not deteriorate during pregnancy [16], fulminant disease may still require urgent surgical intervention. The optimal surgical management of severe or fulminant IIH remains a matter of ongoing debate. Available surgical options include optic nerve sheath fenestration, venous sinus stenting, and CSF diversion procedures. Current consensus recommendations primarily support surgical intervention in patients with threatened or progressive visual loss. The choice of intervention depends on the clinical presentation and local expertise [19].

Previous reports have also demonstrated that CSF diversion procedures can be performed safely during pregnancy when indicated [5, 15]. Our case of successful shunting during pregnancy for vision preservation underscores the feasibility of such interventions in experienced centers.

The infrequency of shunting during pregnancy likely reflects the fact that only a small proportion of patients with IIH require surgical treatment overall. Recent multicenter data reported that surgical interventions were necessary in only 3.6% of patients, predominantly due to acute visual deterioration, while surgery for refractory headache alone was associated with limited benefit [4]. These findings emphasize the importance of careful patient selection before considering surgical treatment.

Regarding delivery mode, current guidelines recommend that IIH alone is not an indication for Caesarean section [19, 27]. Assisted deliveries (such as Caesarean section or instrumental deliveries) may be considered if there is a potential for a prolonged second stage of labor in order to reduce Valsalva-associated ICP elevation, but spontaneous delivery is generally well tolerated [14]. Consistent with this, 9 of the 12 pregnancies in our cohort resulted in full-term vaginal births without complications. These observations are consistent with the findings of Vukovic-Cvetkovic et al., who prospectively followed 47 pregnancies in women with IIH. Although the overall Caesarean section rate was 43%, the authors emphasized that the mode of delivery should be determined by obstetric rather than neurological factors and reported no evidence that vaginal delivery adversely affected maternal outcomes. Similar to their findings, the majority of pregnancies in our cohort resulted in uncomplicated vaginal deliveries [28]. Our findings support the recent practical guidance by Thaller et al., which emphasizes individualized obstetric planning without routine surgical delivery [27].

Fertility-related data remain limited in the IIH population. Recent population-based data indicate that women with IIH have lower live birth rates compared to the general population and women with polycystic ovary syndrome, even after adjusting for age and comorbidities. Furthermore the risk for gestational diabetes and preeclampsia was significantly higher in those with IIH [26]. Our study confirms a low rate of post-shunt pregnancies despite the majority of patients being of reproductive age at the time of surgery. This observation might reflect voluntary or medically advised fertility reduction, or subtle disease-related fertility impairment. Notably, some of our patients had undergone prior gynecological procedures (e.g., management of polycystic ovary syndrome), which may have positively influenced reproductive outcomes. Our study also highlights that most patients received shunt implantation while still of reproductive age, consistent with the known epidemiology of IIH. Using an operational definition of reproductive age allows results to be compared across studies, though biological fertility varies individually and declines significantly after age 35 [2].

### Limitations

Interpretation of our results is limited by the retrospective nature of the study and the relatively small number of pregnancies after VP shunt implantation. In addition, detailed ophthalmological findings were not available in a standardized manner for the entire cohort. Our data reflect the experience of a specialized tertiary center for CSF disorders which may not be generalized.

## Conclusions

Our findings suggest that favorable pregnancy outcomes can be achieved in women with IIH who have previously undergone VP shunting with programmable valves and gravitational devices. In our cohort, no recurrence of IIH related symptoms or shunt-related complications was observed during pregnancy or vaginal delivery.

## Data Availability

No datasets were generated or analysed during the current study.

## Abbreviations

BMI: Body mass index
CSF: Cerebrospinal fluid
ICP: Intracranial pressure
IIH: Idiopathic intracranial hypertension
PTCS: Pseudotumor cerebri syndrome
VP: Ventriculoperitoneal
